# Iron homeostasis in full-term, normal birthweight Gambian neonates over the first week of life

**DOI:** 10.1038/s41598-023-34592-z

**Published:** 2023-06-26

**Authors:** James H. Cross, Ousman Jarjou, Nuredin Ibrahim Mohammed, Santiago Rayment Gomez, Bubacarr J. B. Touray, Noah J. Kessler, Andrew M. Prentice, Carla Cerami

**Affiliations:** 1 Medical Research Council Unit The Gambia at the London, School of Hygiene & Tropical Medicine, Atlantic Boulevard, Fajara, P.O. Box 273, Banjul, The Gambia; 2 Cardiff University, Cardiff, CF10 3AT Wales UK; 3 Department of Genetics, University of Cambridge, Cambridge, CB2 3EH UK

**Keywords:** Neonatology, Homeostasis, Paediatric research, Nutrition, Physiology, Innate immunity

## Abstract

Human neonates elicit a profound hypoferremia which may protect against bacterial sepsis. We examined the transience of this hypoferremia by measuring iron and its chaperone proteins, inflammatory and haematological parameters over the first post-partum week. We prospectively studied term, normal weight Gambian newborns. Umbilical cord vein and artery, and serial venous blood samples up to day 7 were collected. Hepcidin, serum iron, transferrin, transferrin saturation, haptoglobin, c-reactive protein, α1-acid-glycoprotein, soluble transferrin receptor, ferritin, unbound iron-binding capacity and full blood count were assayed. In 278 neonates we confirmed the profound early postnatal decrease in serum iron (22.7 ± 7.0 µmol/L at birth to 7.3 ± 4.6 µmol/L during the first 6–24 h after birth) and transferrin saturation (50.2 ± 16.7% to 14.4 ± 6.1%). Both variables increased steadily to reach 16.5 ± 3.9 µmol/L and 36.6 ± 9.2% at day 7. Hepcidin increased rapidly during the first 24 h of life (19.4 ± 14.4 ng/ml to 38.9 ± 23.9 ng/ml) and then dipped (32.7 ± 18.4 ng/ml) before rising again at one week after birth (45.2 ± 19.1 ng/ml). Inflammatory markers increased during the first week of life. The acute postnatal hypoferremia in human neonates on the first day of life is highly reproducible but transient. The rise in serum iron during the first week of life occurs despite very high hepcidin levels indicating partial hepcidin resistance.

**Trial Registration**: clinicaltrials.gov (NCT03353051). Registration date: November 27, 2017.

## Introduction

During pregnancy, the mother increases iron absorption and turnover of erythrocytes to provide for the growing fetus^1^. As maternal hepcidin decreases during the third trimester, placental iron transfer rises^2^. This leads to higher cord blood TSAT and serum iron levels compared to those of the mother at delivery^3–5^, even in anaemic mothers^6^. To protect the fetus against possible iron overload during the last trimester, fetal-derived hepcidin regulates iron transfer via degradation of ferroportin on placental syncytiotrophoblasts^7^. As a result, umbilical cord hepcidin concentrations of term neonates are higher than those of the mother before and during delivery^8–10^.

In babies born at term, cord levels of IL-6 increase fourfold even in the absence of evident infection^11^. Since the placenta is impermeable to IL-6^11,12^, these high cord blood levels indicate that labour is associated with a fetal-neonatal inflammatory response, potentially triggered by labour-related mechanisms or exposure to infectious agents. Immediately after delivery, newborns face the most complex multi-organ physiological adaption that they will ever experience. Increased IL-6 levels in the newborn are thought to assist with organ system transition at birth (e.g. cytokine-induced synthesis of lung surfactant proteins^13^) and the activation of the immune system in the newborn^14^. IL-6 also activates the JAK-STAT pathway, leading to the induction of hepcidin synthesis^15^. In previous studies, we^16,17^ and others^18–21^ have demonstrated a rapid and profound hypoferremia occurring within the first few hours after delivery. This is assumed to have evolved as a defence against early-onset neonatal sepsis (EONS) and remains robust in premature and low-birthweight babies^17^. Several studies have shown that postnatal peripheral hepcidin and prohepcidin (precursor) levels are higher than those in cord blood^9,18,19^. We previously observed an initial increase in hepcidin within the first 12 h of life in healthy newborns, positively correlated to raised IL-6 levels^16^, and we confirmed this in low birth weight and premature newborns^17^. Our data suggested that the IL-6/hepcidin/ferroportin axis plays a partial, but probably not exclusive, role in orchestrating the hypoferremia.

Here, we examine the duration of the hypoferremia and the likely regulatory influences in normal term babies by analysing serial blood samples over the first week of life.

## Results

**Figure 1:**
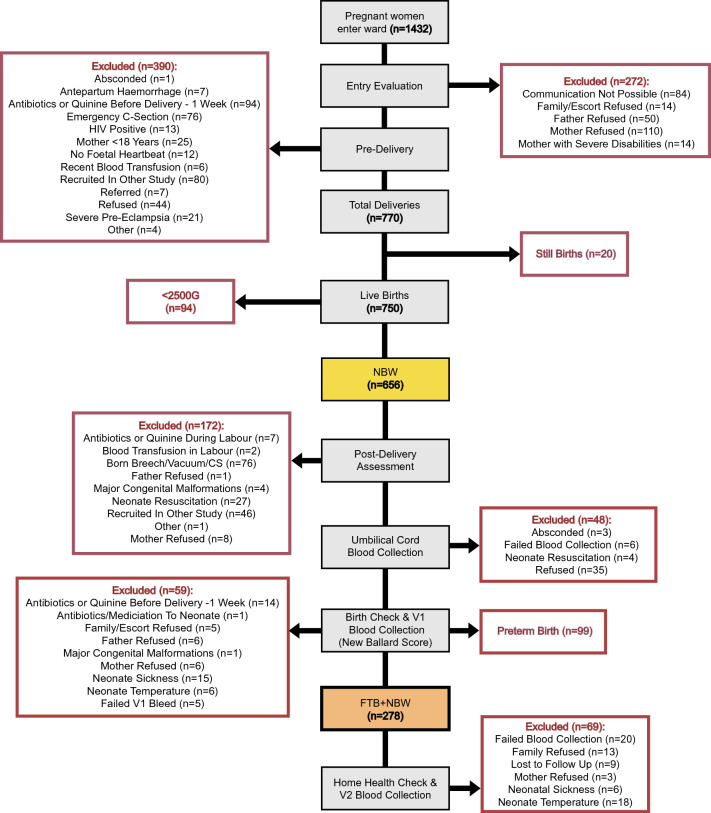
CONSORT diagram for participant flow. 278 full-term (FTB), normal birthweight (NBW) neonates were recruited to the study. Red outlined boxes represent all neonates excluded.

A CONSORT diagram summarising subject recruitment is shown in Fig. 1. There were 278 neonates with paired umbilical cord blood taken at birth and venous blood samples taken in the first 6–24 h after birth (V1 samples). Of these, 224 provided a second venous blood sample during the first week of life (V2, V3 or V4).

### Neonatal characteristics

Baseline characteristics are shown in Table 1. Newborns were healthy vaginally delivered babies, with a mean gestational age of 39.4 ± 1.3 wk and a mean birthweight of 3299 ± 368 g. Many mothers (81.7%) received iron and folic acid during pregnancy as per WHO guidelines. Mean anthropometric measurements of all neonates fell within the 25th and 75th centiles of the WHO growth charts for gestational age^22^. Birthweight increased by 81 ± 7 g/wk and girls averaged 46 ± 8 g lighter than boys. There was no association between sex and gestational age. The mean times after birth of the bleeds were: V1 = 12.7 ± 0.32 h; V2 = 57.6 ± 15.6 h; V3 = 105.8 ± 17.0 h; and V4 = 156.7 ± 21.0 h.

**Table 1:** Demographic, clinical and pregnancy outcome characteristics of the mothers and their newborns.

Study group	Exclusions	V1 (total sample)	V2	V3	V4
Number of participants (n)	54	278	56	100	68
Characteristic	Mean (± SD)				
Gestational age (weeks)	39.4 (± 1.3)	39.4 (± 1.3)	39.4 (± 1.4)	39.3 (± 1.3)	39.6 (± 1.2)
Birth weight (g)	3361.2 (± 377.9)	3299 (± 368.3)	3270.4 (± 355.3)	3293.1 (± 364.7)	3281.9 (± 378.7)
Head circumference (cm)	34.5 (± 1.0)	34.6 (± 1.0)	34.5 (± 1.0)	34.6 (± 1.0)	34.6 (± 1.2)
Length (cm)	50.0 (± 1.6)	50.0 (± 1.6)	50.2 (± 1.5)	50.0 (± 1.5)	49.9 (± 1.7)
Maternal hemoglobin ≤ 7 days before delivery (g/dl)	11.6 (± 2.2)	11.6 (± 1.8)	11.4 (± 1.8)	11.8 (± 1.6)	11.6 (± 1.8)
Age of mother (years)	27.5 (± 6.8)	29.7 (± 6.9)	31.2 (± 7.3)	28.9 (± 6.5)	31.4 (± 6.8)
1 min APGAR Score (0–10)	9.5 (± 0.8)	9.6 (± 0.8)	9.6 (± 0.7)	9.6 (± 0.9)	9.6 (± 0.7)
Time from aamission to birth (hours)	3.3 (± 4.5)	3.2 (± 5.2)	3.7 (± 5.7)	2.7 (± 4.1)	3.5 (± 6.6)
Time from delivery to V1 blood collection (hours)	13.1 (± 5.6)	12.7 (± 5.4)	12.3 (± 5.7)	13.1 (± 5.0)	12.1 (± 5.4)
Time from delivery to V2 blood collection (hours)	–	57.2 (± 15.6)	57.6 (± 15.6)	–	–
Time from delivery to V3 blood collection (hours)	–	105.8 (± 17.0)	–	105.8 (± 17.0)	–
Time from delivery to V4 blood collection (hours)	–	156.7 (± 21.0)	–	–	156.7 (± 21.0)
	Percentage % (n)				
Percentage male (%)	55.5% (30)	54.3% (151)	46.4% (26)	58.0% (58)	54.4% (37)
G6PD Deficiency positive (%)	14.8% (8)	11.5% (32)	8.9% (5)	10.0% (10)	13.2% (9)
Multiple births (%)	0.0% (0)	2.2% (6)	1.8% (1)	4.0% (4)	1.5% (1)
Percentage of mother on antenatal iron/folic acid (%)	81.5% (44)	81.7% (227)	83.9% (47)	80.0% (80)	82.3% (56)

Data are presented as arithmetic mean (± SD) or as a proportion (%). Exclusions represent newborns successfully bled at cord and V1 timepoints but who were lost to follow up or sick before V2-4 blood draw was completed.

### Changes in iron and chaperone proteins in the first week of life

**Figure 2:**
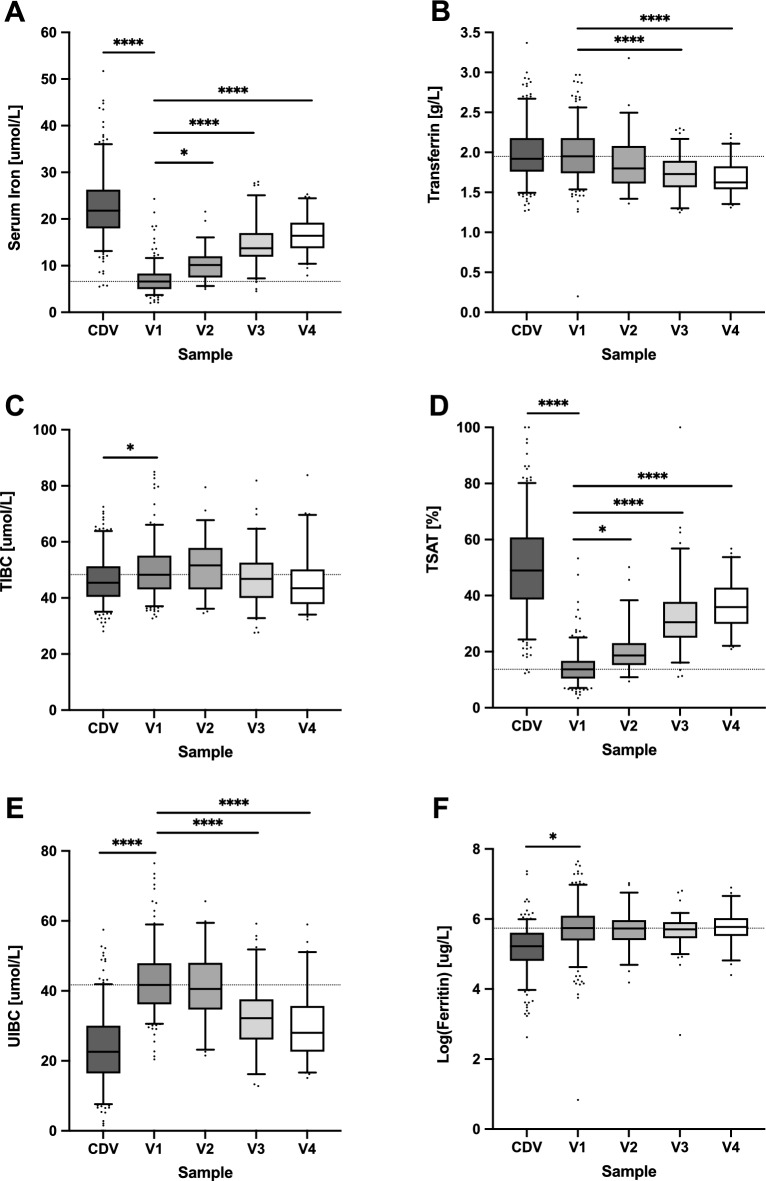
Changes in iron and chaperone proteins in the first week of life. Boxplots showing serum iron (**A**), transferrin (**B**), TIBC (**C**), TSAT (**D**), UIBC (**E**), and ferritin (**F**) between CDV (cord blood), V1 (≥ 6- < 24 h) and V2 (≥ 24- < 80 h), V3 (≥ 80—< 136 h) or V4 (≥ 136- < 192 h) for each individual. Top and bottom 5th percentile are shown as outliers. Repeat-measures ANOVA was conducted between V1 vs V2, V3 and V4 groups. **** = *P* < 0.0001, *** = *P* < 0.001, ** = *P* < 0.01, * = *P* < 0.05.

Iron metabolism parameters over the first week of life are shown in Fig. 2 and Supplemental Table 1. Following the acute hypoferremia on Day 1 (CDV serum iron = 22.7 ± 7.0 µmol/L vs. V1 = 7.3 ± 4.6 µmol/L, *P* < 0.0001), there was a steady increase in serum iron to 16.5 ± 3.9 μmol/L by the final visit at one week of life (V4 vs. V1, *P* < 0.0001) (Fig. 2A and Supplemental Table 1). There was a slight but significant decrease in transferrin levels (Fig. 2B and Supplemental Table 1) but TIBC levels remained relatively constant (Fig. 2C and Supplemental Table 1), and hence TSAT levels mirrored those for serum iron with a very sharp decline between cord blood and V1 (CDV = 50.2 ± 16.7% vs. V1 = 14.4 ± 6.1%, *P* < 0.0001) followed by a marked increase over subsequent days to 36.6 ± 9.2% at V4 (*P* < 0.0001) (Fig. 2D and Supplemental Table 1). UIBC consequently decreased during the first week of life (from V1 = 44.1 ± 18.0 μmol/L to V4 = 29.6 ± 9.7 μmol/L, *P* < 0.0001) (Fig. 2E and Supplemental Table 1). Ferritin levels were very high on Day 1 of life (V1 = 393 ± 312.6 μg/L) and remained constant over the first week of life (Fig. 2F and Supplemental Table 1).

### Changes in hepcidin and inflammatory markers in the first week of life

**Figure 3:**
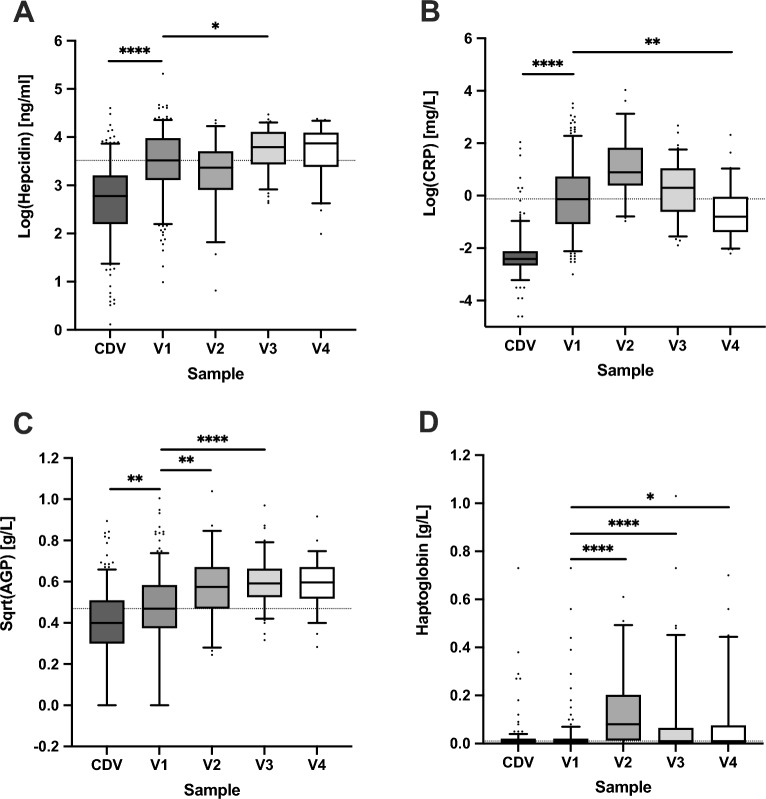
Changes in hepcidin and inflammatory markers in the first week of life. Boxplots showing hepcidin (**A**), CRP (**B**), AGP (**C**), and haptoglobin (**D**) between CDV (cord blood), V1 (≥ 6- < 24 h) and V2 (≥ 24- < 80 h), V3 (≥ 80—< 136 h) or V4 (≥ 136- < 192 h) for each individual. Top and bottom 5th percentile are shown as outliers. Repeat-measures ANOVA was conducted between V1 vs V2, V3 and V4 groups. **** = *P* < 0.0001, *** = *P* < 0.001, ** = *P* < 0.01, * = *P* < 0.05.

Hepcidin increased rapidly during the first 24 h of life (19.4 ± 14.4 ng/ml to 38.9 ± 23.9 ng/ml, *P* < 0.0001) and remained rather constant with a small rise at one week after birth (45.8 ± 19.1 ng/ml) (Fig. 3A and Supplemental Table 1). CRP levels were low in cord blood (median 0.09 mg/L; IQR, 0.07–0.12) and increased nearly tenfold 6–24 h after birth (median 0.88 mg/L; IQR, 0.34–2.08 at V1, *P* < 0.0001) followed by a further rise to a median of 2.44 mg/L; IQR, 1.47–6.18 at V2 (*P* < 0.0001) and then declined by the end of the first week of life (V4 = median 0.45 mg/L; IQR, 0.25–0.96, *P* < 0.0001 vs. V2) (Fig. 3B and Supplemental Table 1). AGP showed a small increase after birth and then a further slow rise during the first week of life (Fig. 3C and Supplemental Table 1). The haem-binding inflammatory-response protein, haptoglobin, increased between V1 and V2 timepoints (from 0.03 ± 0.07 g/L to 0.1 ± 0.2 g/L, *P* < 0.0001) and then declined slightly over the first week of life (V4 = 0.08 ± 0.2 g/L) (Fig. 3D and Supplemental Table 1).

### Changes in haematological indices in the first week of life

**Figure 4:**
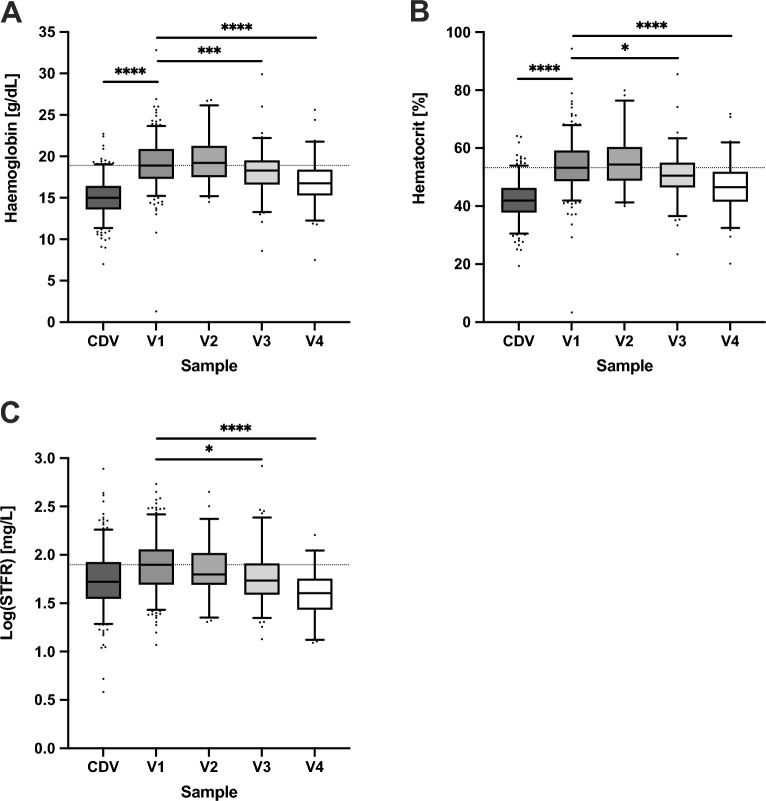
Changes in haematological indices in the first week of life. Boxplots showing the difference in haemoglobin (**A**), haematocrit (**B**), and sTfR (**C**) between CDV (cord blood), V1 (≥ 6- < 24 h) and V2 (≥ 24- < 80 h), V3 (≥ 80—< 136 h) or V4 (≥ 136- < 192 h) for each individual. Top and bottom 5th percentile are shown as outliers. Repeat-measures ANOVA was conducted between V1 vs V2, V3 and V4 groups. **** = *P* < 0.0001, *** = *P* < 0.001, ** = *P* < 0.01, * = *P* < 0.05.

Cord haemoglobin (15.1 ± 2.3 g/dl) and haematocrit (42.2 ± 7.0%) were high, and there was a further increase by V1 (haemoglobin to 19.1 ± 2.9 g/dl, haematocrit to 53.9 ± 8.9%, both *P* < 0.0001). This was followed by a steady decline back towards cord-like levels by V4 (haemoglobin to 16.9 ± 3.0 g/dl, *P* < 0.0001 and haematocrit to 46.9 ± 9.1%, *P* < 0.0001 vs. V1) (Fig. 4A, B and Supplemental Table 1). Soluble transferrin receptor levels declined steadily over the first week, reflecting the fact that there was a net breakdown of haemoglobin during the first week of life and an abundance of circulating iron to supply the needs of any ongoing erythropoiesis (Fig. 4C and Supplemental Table 1).

Weighted Pearson correlation network analysis between the iron and inflammation markers in all samples (CDV, V1 and V2-4) are shown in Supplemental Fig. 1. The unconnected nodes for birthweight and gestational age reflect their lack of influence on any of the markers in postnatal blood. As expected, there were consistent associations between serum iron, TSAT, UIBC and TIBC at all time points, but the V1 nodes were notably separated from CDV and V2-V4, underscoring the unusual nature of the immediate postnatal hypoferremia. Haemoglobin and haematocrit followed a similar pattern, with cord values only correlating to V2-V4 values. The inflammatory markers CRP, AGP and haptoglobin were generally associated as would be expected (see the lower part of the network), but ferritin, also an inflammatory marker, was notably separate.

### Comparisons of iron and inflammation markers in arterial (CDA) and venous cord blood (CDV)

Supplemental Fig. 2 illustrates the comparisons between iron and inflammation markers in venous and arterial cord blood. For some of the markers there was a signifcant difference, however the magnitude of the differences were minimal. TSAT was slightly higher in venous blood (50.5 ± 16.5%) than arterial cord blood (46.9 ± 15.6%, *P* < 0.0001). Conversely, arterial blood had higher ferritin (277.0 ± 235.0 vs. 215.7 ± 161.1 µg/L, *P* < 0.0001), UIBC (27.1 ± 11.2 vs. 23.4 ± 10.3 µmol/L, *P* < 0.0001) and TIBC (49.8 ± 9.1 vs. 46.1 ± 8.1 µmol/L, *P* < 0.0001). There were limited differences in red blood cell indices between venous and arterial cord blood, but white cell counts (except for granulocyte count and percentage) were significantly higher in arterial blood (Supplemental Table 2). Conversely, platelet counts were significantly higher in venous blood.

### Associations between hepcidin, TSAT and serum iron

**Figure 5:**
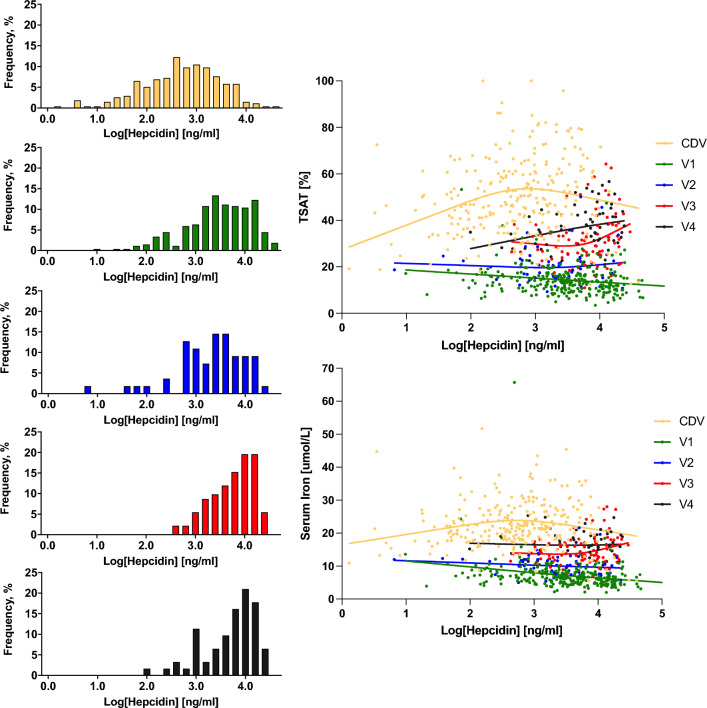
Changes in hepcidin levels and associations with serum iron and TSAT in the first week of life. Left-hand panels: Distributions of log-hepcidin levels at each time point. Right-hand panels: Scatterplots of TSAT and serum iron against log-hepcidin. Lines indicate the best fit from quadratic regression. TSAT vs Log-hepcidin: CDV R^2^ = 0.068, 246 degrees of freedom (DF), V1 R^2^ = −0.037, 265 DF, V2 R^2^ = −0.003, 51 DF, V3 R^2^ = −0.050, 86 DF, V4 R^2^ = −0.084, 56 DF. Serum iron vs log-hepcidin: CDV R^2^ = 0.035, 251 DF, V1 R^2^ = −0.056, 265 DF, V2 R^2^ = −0.019, 51 DF, V3 R^2^ = −0.037, 88 DF, V4 R^2^ = −0.000, 58 DF.

In cord blood there was no detectable association between log-hepcidin and circulating iron levels assessed either as TSAT or serum iron (Fig. 5). In the first post-partum sample (V1) there was a modest inverse association between hepcidin and both TSAT (R^2^ = −0.037, 265 degrees of freedom) and serum iron (R^2^ = −0.056, 265 degrees of freedom). As hepcidin levels increased from V2 the association was attenuated.

### Influence of duration of pregnancy on cord blood iron markers

Despite, in this study of term infants, having data for only 6 separate gestational weeks (37–42 weeks with 2 additional babies estimated at 43 weeks) there were strong associations between iron markers and gestational age. As the duration of pregnancy lengthened transferrin, and hence TIBC, increased (*P* = 0.001 and < 0.001) as did sTfR (*P* = 0.0025). Serum iron declined (*P* = 0.01) and TSAT declined very markedly; by 3% per week (*P* < 0.0001). UIBC increased markedly (*P* < 0.0001).

## Discussion

This study reconfirms our previous observation of a rapid and acute hypoferremia in the first day of life^16,17^ with serum iron dropping from 23 to7 µmol/L and TSAT falling from 50 to 14%. This may have evolved as an arm of innate immunity designed to protect from neonatal septicemia, a common cause of neonatal death. We now show that this is a transient effect with serum iron and TSAT steadily increasing over the first week of life.

Our prior analyses^16,17^ using data from independent studies, revealed that the early hypoferremia was, at least in part, likely driven by an inflammatory response to the birth process eliciting a rapid IL-6-mediated rise in hepcidin. Hepcidin blocks the release of iron from enterocytes and macrophages^1^ and thereby reduces serum iron through the dual actions of preventing iron absorption and recirculation. In neonates, who receive insignificant amounts of dietary iron on Day 1, the latter mechanism dominates, and the hypoferremia represents a temporary redistribution of iron away from the extracellular plasma where it would enhance the growth of any ingressing bacteria or fungi^16^.

**Figure 6:**
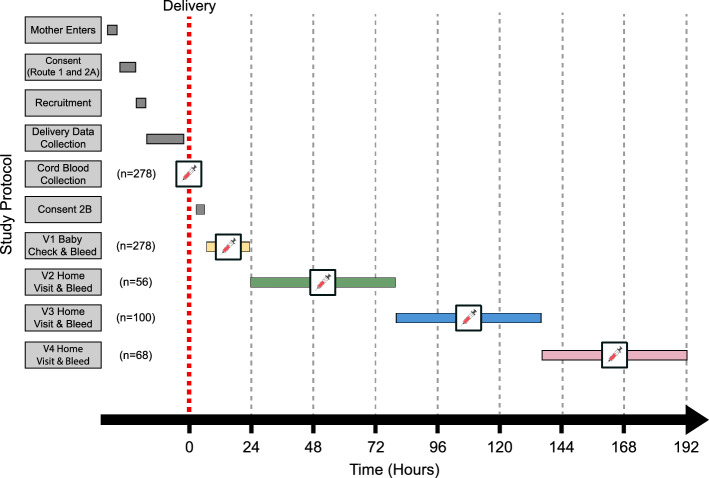
Study recruitment and blood draw design. Mothers were approached on entering the Kanifing General Hospital (KGH) maternity ward, The Gambia. This was followed by the consenting process, recruitment and delivery data collection. At delivery, venous (CDV) and arterial (CDA) cord blood was collected after one minute delayed cord clamping. The neonate was weighed after cord blood collection. At 6–24 h post-delivery, the research study clinician conducted a health check of the mother and newborn. New Ballard Score was used to establish gestational age. If the neonate was deemed healthy, a V1 blood draw was completed. Follow-up in the community was conducted 24–216 h post-delivery. This involved a health check of the mother and newborn by the study nurse. If deemed healthy, the newborn was bled again at one other timepoint (V2: ≥ 24 h—< 80 h, or V3: ≥ 80 h—< 136 h, or V4: ≥ 136—< 192 h).

Cross-sectional associations between homeostatic hormones (such as insulin, leptin and hepcidin) and their target metabolites are frequently hard to interpret. Steady-state associations are generally positive; when the metabolite is above its target level the hormone increases to effect a correction. The reverse is the case in the short term; raised hepcidin elicits a hypoferremia. On the first postnatal day there is a rapid rise in hepcidin which acutely suppresses serum iron and there is a negative correlation between hepcidin and iron (Fig. 6). A surprising element of the current data is that iron and TSAT levels start to revert to normal despite hepcidin levels continuing to rise over the first week, reaching values three– to fourfold higher than those observed in healthy adults^23^. Furthermore, there was no correlation between hepcidin and serum iron or TSAT during the first week of life (V2-4 samples). This unexpected disconnect between the relatively high levels of serum iron and TSAT coupled with high hepcidin concentrations suggests that early neonatal iron metabolism is desensitised to the action of hepcidin. Thus, the sequestration of intracellular iron apparent on Day 1 is not maintained. This could be because the intracellular iron pools are saturated in the early post-partum period. We hypothesise that macrophage cellular iron pools are increased in the first hours of life, initially due to the physiological haemolysis of fetal erythrocytes^24^, followed by the uptake of transferrin-iron complexes^25^. Erythrophagocytosis and the recycling of fetal haemoglobin by haem oxygenase also add to intracellular iron levels^26^. This is further exacerbated by the effects of inflammation-induced hepcidin excess at 6–24 h post-delivery, leading to hepcidin-induced co-degradation^27^ and/or hepcidin occlusion^28^ of the transmembrane iron transporter, ferroportin. We propose that the initial hepcidin levels reduce expression of ferroportin on macrophage cell membranes, thus eliciting the immediate postnatal hypoferremia, but that complete removal of all ferroportin molecules from the cell membrane is not achieved. This is supported by in vitro hepcidin challenge experiments showing a halving of ferroportin expression within 4 h^29^, but that complete removal of ferroportin was not achieved. Previous authors have suggested that excess levels of hepcidin more likely result in blocking the central cavity of ferroportin rather than its internalisation^30^.

Other studies have shown that following the neonatal period, circulating hepcidin levels decline to levels similar or lower to those observed in cord blood ^31–33^. Increased expression of growth factors (IGF-1, HGF, EGF, PDGF-BB) is thought to cause the downregulation of hepcidin transcription^34,35^. This study shows that this trend does not begin until after the first week of life.

CRP levels peaked between 24 and 80 h post-delivery, with again a surprising absence of correlation with hepcidin concentrations. This is despite the well-documented regulatory pathways of infection and inflammation on iron regulation^36^. Previous studies have suggested that the lack of correlation between hepcidin, IL-6 and CRP is due to differences in the kinetics of the molecules involved. IL-6 concentrations spike very early in the course of infection or inflammation, followed by an increase in hepcidin, then a rise in CRP and finally the release of AGP^37^.

The great majority of mothers in this study reported that they received iron and folic acid in pregnancy as per Gambian government guidelines, but 52% remained anaemic in the last week before delivery. Despite this, ferritin levels in cord blood were high (CDV: 213 ± 158 µg/L) and levels almost doubled immediately after delivery to 394 ± 313 µg/L in neonates at V1. It has previously been suggested that this is due to the physiological hemolysis of fetal red blood cells, which contain ferritin in high concentrations^21^. Similarly, we found elevated levels of haptoglobin, peaking at 0.1 ± 0.2 g/L at 24–80 h of life. We suggest this is another layer of nutritional immunity, as haptoglobin binds to haemoglobin, further restricting iron availability to invading microorganisms^38^.

We undertook the comparative analysis of iron markers and inflammation in arterial and venous cord blood to ensure that non-standardised sampling of ‘cord blood’ in prior studies did not affect the comparisons between cord and postnatal bloods. Serum iron was identical between the cord blood vessels, and although there were significant differences for TIBC and hence reciprocally for TSAT, the differences were only on the order of 6–7%. Transferrin levels were similar, suggesting that differences in non-transferrin iron-binding compounds account for the slight difference in TIBC and TSAT.

Current understanding is that fetal iron is primarily accrued in the third trimester of pregnancy; a process aided by down regulation of maternal hepicidin and upregulation of placental iron transporters^39^. Our data strongly suggest that after 37 weeks the growth demands of the fetus (81 g/week) are out-stripping the ability of the placenta to supply iron, at least in the Gambian setting where mothers tend to be somewhat iron deficient. This conclusion is based upon the marked increases in transferrin and sTfR and decreases in serum iron and TSAT from 37 weeks onwards.

There are several strengths and limitations to our study. The sample size and the relative homogeneity of responses across most analytes provide confidence in the trends observed. A limitation is that maternal iron markers in mid-gestation, parturition and after delivery were not measured. This would have provided information as to what effect maternal iron and inflammation status had on the neonatal iron marker fluctuations we studied. Additionally, asymptomatic chorioamnionitis was not excluded in the mothers. Measurement of pro-inflammatory cytokines (e.g. IL-6 and IL-22) and growth factors (IGF-1, HGF, EGF and PDGF-BB) may have provided additional insights into the regulation of postnatal iron metabolism, but cost and sample volume constraints precluded their inclusion. Study design was significantly shaped by the necessity to minimise the burden on participants; as a result, the persistence of hypoferremia was not assessed beyond the one-week observation period. Variables governing the hepcidin-independent regulation of iron redistribution are not yet known, so could not be measured in our study. The effects of diurnal rhythm, iron supplementation and infection were not assessed and could be a direction for future research.

In conclusion, our results suggest that early postnatal hypoferremia is a fast-acting yet short-lived adaptation. This is followed by a period of hepcidin desensitisation as iron efflux into the serum continues even in the presence of high serum hepcidin concentrations. The reduced need for iron for erythropoiesis during the first week of life could also result in increased serum iron concentration. This interpretation is supported by the observed decrease in sTfR levels over the first week, indicating that erythroid tissues were not demanding iron. We have previously proposed that, in principle, the duration of postnatal hypoferremia might be extendable through the administration of mini-hepcidins as an ancillary tool against antimicrobial-resistant infections^17^. The new data presented here suggest that any such intervention would need to overcome or circumvent the hepcidin resistance we report in the first week of life.

## Subjects and methods

Full details of the NeoInnate Study (clinicaltrials.gov, NCT03353051) can be found in the published protocol paper^40^.

### Study design

The NeoInnate Study tested whether preterm and/or low birthweight babies were capable of inducing the acute hypoferremia previously noted in full-term babies. Results for the primary outcomes have been presented elsewhere^17^. Here we describe the pre-planned secondary analysis of longitudinal changes in iron, haematological and inflammatory parameters over the first week of life within the term, normal-weight babies from the control group. All babies were sampled from the cord blood artery (CDA) and vein (CDV) and had an early postnatal draw (V1) at 6–24 h. For the longitudinal analysis over the first week of life and to avoid more than two blood draws per baby, the babies were then randomly allocated for a second blood draw at ≥24–<80 h (V2), ≥80–<136 h (V3) or ≥136–<192 h (V4) (Fig. 6). Data collection started on the 5th July 2017 and ended on 1st February 2019.

### Ethics, standards and informed consent

The trial was approved by the Medical Research Council Unit The Gambia at London School of Hygiene and Tropical Medicine (MRCG at LSHTM) Scientific Coordinating Committee, the Joint Gambia Government/MRC Ethics Committee (no. SCC1525) and the London School of Hygiene and Tropical Medicine Ethics Committee (no. 14316)^40^ and conducted according to Good Clinical Practice (GCP) standards. The study procedures were explained to the neonate's mother/guardians orally and in writing. A neonate was only recruited into the study after the written informed consent was provided by the mother/guardian.

### Study setting

Study participants were recruited from Kanifing General Hospital (formerly Serrekunda General Hospital) in the urban Kanifing region of The Gambia, West Africa.

### Recruitment, screening and enrolment

We enrolled 300 neonates into this longitudinal arm of the NeoInnate Study. For inclusion in this arm of the study, neonates were healthy, medically stable (not requiring resuscitation and with no signs of sepsis) with a gestational age ≥ 37 completed weeks (assessed by New Ballard Score^41^) and weighed ≥ 2500 g. None of the neonates received IV fluids during the study period.

Pregnant mothers were excluded from the study if they were below the age of 18 years, had no fetal heartbeat detected upon admission, were known to be HIV-positive, received anti-tuberculosis treatment, had taken antibiotics in the last seven days, had a blood transfusion in the previous month, were suffering from severe pre-eclampsia or antepartum haemorrhage, or were in another research study.

Babies were excluded at the delivery stage for the following reasons: major congenital malformations (not including polydactylism), blood transfusions given to mother or neonate, severe birth asphyxia (requiring resuscitation), neonates born via breech, vacuum or caesarean section.

After the delivery stage, babies were excluded following the detection of infection or illness (information gained from venous bleed or review of systems). Neonates were also removed from the study protocol if any medication other than intramuscular vitamin K, tetracycline eye ointment or immunisations was given. All medications given to mothers and neonates during the study period were recorded. Mothers who delivered multiple newborns were invited to enrol one of their neonates into the study.

### Sample collection

Once the neonate was fully delivered, one-minute delayed cord clamping was used (following World Health Organisation (WHO) policy^42^). The umbilical cord was separated from the baby and the placenta. A trained study nurse cleaned the cord and identified the umbilical arteries (CDA) and umbilical vein (CDV). Blood was collected from each using separate blood draw equipment.

At 6–24 h post-delivery, recruited mothers and their neonates were invited to a private consultation with the study research clinician. Demographic data were collected, along with a complete review of systems of the mother and neonate, and newborn anthropometry. Neuromuscular and physical maturation of each neonate was assessed using the New Ballard Score^41^. Immediately after passing the health assessment, a 3.5 ml venous blood draw was performed on all neonates (V1).

During the community visit at the home of the neonate, a review of systems in the mother and child were conducted by a research nurse. This was followed by collecting data on medication, behaviour and immunisations of the neonate after leaving the hospital. A further sample of 3.5 ml venous blood was then collected (V2-4) if the neonate was healthy.

### Laboratory analyses

A full haematology panel (using a Medonic M20M GP, Boule Diagnostics, Spanga, Sweden) and glucose-6-phosphate dehydrogenase deficiency test (R&D Diagnostics Limited, Papagos, Greece) were conducted on fresh whole blood. Serum was separated and stored at −20 °C prior to analysis of ferritin, iron, unsaturated iron-binding capacity (UIBC), soluble transferrin receptor (sTfR), transferrin, c-reactive protein (CRP), haptoglobin, and alpha-1-acid glycoprotein (AGP) using a fully automated biochemistry analyser (Cobas Integra 400 plus, Roche Diagnostic, Switzerland). Transferrin saturation (TSAT) was calculated. Serum samples were assessed for hepcidin concentration by ELISA (hepcidin-25 (human) EIA Kit, DRG, USA) with a dynamic range of 0.135–81 ng/mL.

To ensure a consistent assessment of haemolysis in all serum samples, batches of samples were thawed before entering the biochemistry analyser and visually scored by a single operator. A previously published specimen integrity chart for haemolysis was used as a reference^43^. Samples were scored 0 (yellow; 0 g/L) to 6 (dark red; 8 g/L). Samples scoring ≥ 5 were removed from the analysis.

### Sample size determination

Sample size calculations for the primary outcomes of the NeoInnate Study^17^ were based on data from a previous study^16^ and are summarised in the protocol paper^40^. The secondary outcomes presented here were not subjected to a formal sample size analysis.

### Statistical analysis

Statistical analysis and preparation of figures were conducted using STATA v15.1 (Stat-Corp LP, College Station, TX, USA), DataDesk version 7.0.2 (Data Description Inc) and GraphPad Prism (GraphPad Software INC, CA 92,037, USA). For continuous variables, baseline characteristics are presented as means (± SD) for normally distributed variables. All skewed data (hepcidin, CRP, AGP, sTfR and ferritin) were transformed. Categorical variables are reported as proportions (%). Trends over time and differences between timepoints were assessed using repeat measures ANOVA and reported as Scheffé’s post hoc statistics. The proportion of missing data for the key variables analysed was small (< 5%); thus, we did not impute missing data. Comparisons of iron and inflammation markers in arterial (CDA) and venous cord blood (CDV) were conducted using two-sided paired t-tests. Weighted Pearson network analysis was conducted using the “Network App” Shiny application (https://github.com/JolandaKossakowski/NetworkApp). The network was formatted using the Fruchterman-Reingold Algorithm^44^, only showing moderate or strong (> 0.3) associations between nodes. V2, V3 and V4 nodes were combined to aid visualisation.

## Supplementary Information

Supplementary Information.

## Data Availability

All data will be made available to researchers upon reasonable request to the study PI and clearance by the MRCG Scientific Coordinating and Ethics Committees.
