# Supplementary material for: Iron homeostasis in full-term, normal birthweight Gambian neonates over the first week of life
Source: Sci Rep. 2023 Jun 26;13:10349. doi: 10.1038/s41598-023-34592-z (PMC10293170; doi:10.1038/s41598-023-34592-z)
Supplement: Supplementary file 1 — Supplementary Information. [file 41598_2023_34592_MOESM1_ESM.docx]

**SUPPLEMENTAL INFORMATION**

**Title**

**Iron homeostasis in full-term, normal birthweight Gambian neonates over the first week of life.**

**Authors**

James H. Cross^1^, Ousman Jarjou^1^, Nuredin Ibrahim Mohammed^1^, Santiago Rayment Gomez^2^, Bubacarr J.B Touray^1^, Noah J. Kessler ^3^, Andrew M. Prentice^1^, Carla Cerami^1*^.

**Supplemental Table 1: Comparison of iron status, inflammation and haematological parameters in cord and postnatal samples.**

| **Sample Type** | **n** | **CDV** | **n** | **V1** | **n** | **V2** | **n** | **V3** | **n** | **V4** | ***V1 vs V2***  ***(P value)*** | ***V1 vs V3***  ***(P value)*** | ***V1 vs V4***  ***(P value)*** |
| --- | --- | --- | --- | --- | --- | --- | --- | --- | --- | --- | --- | --- | --- |
| Serum Iron (µmol/L) | 275 | 22.7 (±7.0) | 271 | 7.3 (±4.6) | 52 | 10.0 (±3.3) | 91 | 14.8 (±4.6) | 61 | 16.5 (±3.9) | **<0.0001** | **<0.0001** | **<0.0001** |
| TSAT (%) | 273 | 50.2 (±16.7) | 271 | 14.4 (±6.1) | 52 | 20.2 (±8.3) | 89 | 32.6 (±13.0) | 60 | 36.6 (±9.2) | **<0.0001** | **<0.0001** | **<0.0001** |
| Serum Hepcidin (ng/ml) | 277 | 19.4 (±14.4) | 270 | 38.9 (±23.9) | 51 | 32.7 (±18.2) | 90 | 45.9 (±17.9) | 60 | 45.8 (±19.1) | **-** | **-** | **-** |
| Log(Serum Hepcidin) | 277 | 2.7 (±0.8) | 270 | 3.5 (±0.7) | 51 | 3.3 (±0.7) | 90 | 3.7 (±0.4) | 60 | 3.7 (±0.5) | ns | **<0.0001** | ns |
| UIBC (µmol/L) | 273 | 23.7 (±10.4) | 271 | 44.1 (±18.0) | 52 | 41.2 (±10.1) | 89 | 32.2 (±10.2) | 60 | 29.6 (±9.7) | ns | **<0.0001** | **<0.0001** |
| TIBC (µmol/L) | 273 | 46.3 (±8.1) | 271 | 51.4 (±20.7) | 52 | 51.2 (±9.6) | 89 | 46.9 (±9.6) | 60 | 46.1 (±10.6) | ns | ns | ns |
| Serum Ferritin (µg/L) | 275 | 212.6 (±157.7) | 266 | 393.9 (±312.6) | 49 | 335.5 (±206.0) | 91 | 312.4 (±132.3) | 60 | 356.4 (±184.4) | **-** | **-** | **-** |
| Log(Serum Ferritin) | 275 | 5.2 (±0.6) | 266 | 5.7 (±0.7) | 49 | 5.7 (±0.5) | 91 | 5.7 (±0.5) | 60 | 5.8 (±0.5) | ns | **0.003** | ns |
| Haemoglobin (g/dl) | 270 | 15.1 (±2.3) | 272 | 19.1 (±2.9) | 48 | 19.6 (±2.9) | 93 | 18.2 (±2.9) | 63 | 16.9 (±3.0) | ns | **0.01** | **<0.0001** |
| Haematocrit (%) | 270 | 42.2 (±7.0) | 272 | 53.9 (±8.9) | 48 | 55.3 (±9.6) | 93 | 50.7 (±8.8) | 63 | 46.9 (±9.1) | ns | **0.005** | **<0.0001** |
| Transferrin (g/L) | 275 | 2.0 (±0.3) | 273 | 2.0 (±0.3) | 54 | 1.9 (±0.3) | 91 | 1.7 (±0.2) | 61 | 1.7 (±0.2) | **<0.0001** | **<0.0001** | **<0.0001** |
| Soluble Transferrin Receptor (mg/L) | 273 | 6.0 (±2.0) | 271 | 6.9 (±2.2) | 52 | 6.6 (±2.0) | 90 | 6.1 (±2.2) | 61 | 5.1 (±1.2) | **-** | **-** | **-** |
| Log(Soluble Transferrin Receptor) | 273 | 1.7 (±0.3) | 271 | 1.9 (±0.3) | 52 | 1.8 (±0.3) | 90 | 1.8 (±0.3) | 61 | 1.6 (±0.3) | **<0.0001** | **<0.0001** | **<0.0001** |
| Haptoglobin (g/L) | 275 | 0.02 (±0.1) | 273 | 0.03 (±0.07) | 54 | 0.1 (±0.2) | 91 | 0.08 (±0.2) | 61 | 0.08 (±0.2) | **<0.0001** | **0.0002** | ns |
| Serum AGP (g/L) | 275 | 0.2 (±0.1) | 273 | 0.3 (±0.2) | 54 | 0.4 (±0.2) | 91 | 0.4 (±0.1) | 61 | 0.4 (±0.1) | **-** | **-** | **-** |
| Sqrt(Serum AGP) | 275 | 0.4 (±0.2) | 273 | 0.5 (±0.2) | 54 | 0.6 (±0.2) | 91 | 0.6 (±0.1) | 61 | 0.6 (±0.1) | **<0.0001** | **<0.0001** | **<0.0001** |
| Serum CRP (mg/L) | 273 | 0.2 (±0.7) | 273 | 2.3 (±4.1) | 53 | 5.0 (±8.3) | 91 | 2.0 (±2.3) | 61 | 0.9 (±1.5) | **-** | **-** | **-** |
| Log(Serum CRP) | 273 | -2.3 (±0.8) | 273 | -0.1 (±1.3) | 53 | 1.0 (±1.0) | 91 | 0.2 (±1.0) | 61 | -0.6 (±0.9) | **<0.0001** | ns | **0.007** |
| Mean Corpuscular Volume (fl) | 270 | 97.8 (±6.3) | 272 | 97.7 (±6.0) | 48 | 97.6 (±5.1) | 93 | 96.0 (±5.9) | 63 | 94.1 (±5.7) | ns | **<0.0001** | **<0.0001** |
| Mean Corpuscular Haemoglobin (pg) | 270 | 35.1 (±2.4) | 272 | 34.8 (±2.4) | 48 | 34.8 (±2.1) | 93 | 34.5 (±2.2) | 63 | 34.1 (±2.2) | ns | **<0.0001** | **<0.0001** |
| Mean Corpuscular Haemoglobin Concentration (g/dl) | 270 | 35.8 (±1.0) | 272 | 35.6 (±1.1) | 48 | 35.6 (±1.5) | 93 | 35.9 (±0.9) | 63 | 36.2 (±1.1) | ns | **0.01** | **0.0003** |
| White Blood Cell Count (unit/L) | 269 | 13.3 (±5.7) | 271 | 16.4 (±5.8) | 48 | 10.5 (±5.4) | 92 | 8.8 (±3.1) | 62 | 9.2 (±2.5) | **<0.0001** | **<0.0001** | **<0.0001** |
| Lymphocyte Count (unit/L) | 269 | 4.8 (±3.5) | 271 | 4.2 (±2.9) | 48 | 3.1 (±1.6) | 92 | 3.3 (±1.2) | 62 | 3.5 (±1.0) | **<0.0001** | **0.004** | ns |
| Lymphocyte Percentage (%) | 269 | 35.2 (±7.9) | 271 | 26.5 (±8.8) | 48 | 31.1 (±9.3) | 92 | 38.7 (±7.7) | 62 | 38.5 (±7.1) | **0.005** | **<0.0001** | **<0.0001** |
| MID Cell Count (unit/L) | 269 | 1.04 (±0.6) | 271 | 1.54 (±1.0) | 48 | 1.1 (±0.8) | 92 | 1.2 (±0.8) | 62 | 1.7 (±0.8) | ns | **0.007** | ns |
| MID Cell Percentage (%) | 269 | 8.1 (±2.4) | 271 | 9.8 (±5.6) | 48 | 11.5 (±7.0) | 92 | 14.9 (±7.9) | 62 | 18.8 (±8.2) | ns | **<0.0001** | **<0.0001** |
| Granulocyte Count (unit/L) | 269 | 7.5 (±2.7) | 271 | 10.7 (±4.2) | 48 | 6.3 (±4.0) | 92 | 4.2 (±2.0) | 62 | 4.1 (±1.8) | **<0.0001** | **<0.0001** | **<0.0001** |
| Granulocyte Percentage (%) | 269 | 56.6 (±8.7) | 271 | 63.7 (±8.8) | 48 | 57.4 (±9.3) | 92 | 46.4 (±9.2) | 62 | 42.7 (±9.2) | **0.0001** | **<0.0001** | **<0.0001** |
| Red Blood Cell Count (unit/L) | 270 | 4.3 (±0.7) | 272 | 5.5 (±0.9) | 48 | 5.7 (±1.0) | 93 | 5.3 (±0.9) | 63 | 5.0 (±0.9) | ns | ns | **0.0004** |
| Red Blood Cell Distribution Width (%) | 270 | 15.2 (±1.2) | 272 | 15.4 (±1.4) | 48 | 15.6 (±2.8) | 93 | 15.1 (±0.9) | 63 | 15.1 (±2.2) | ns | **<0.0001** | **<0.0001** |
| Red Blood Cell Distribution Width - Absolute (fl) | 270 | 80.7 (±8.9) | 272 | 82.0 (±8.6) | 48 | 82.6 (±15.9) | 93 | 78.0 (±8.0) | 63 | 75.4 (±7.1) | ns | **<0.0001** | **<0.0001** |
| Platelet Count (unit/L) | 270 | 243.8 (±92.8) | 272 | 258.7 (±94.0) | 48 | 259.4(±91.0) | 93 | 237.6 (±95.4) | 63 | 266.3 (±94.0) | ns | ns | ns |
| Mean Platelet Volume (fl) | 267 | 8.4 (±0.8) | 271 | 8.5 (±0.8) | 47 | 8.5 (±0.9) | 92 | 8.7 (±0.8) | 63 | 9.3 (±0.7) | ns | ns | **<0.0001** |
| Platelet Distribution Width (%) | 267 | 43.5 (±3.0) | 271 | 43.9 (±3.3) | 47 | 44.5 (±3.5) | 92 | 45.4 (±3.6) | 63 | 46.4 (±2.7) | ns | **0.007** | **<0.0001** |
| Platelet Distribution Width - Absolute (fl) | 267 | 11.8 (±1.3) | 271 | 12.1 (±1.4) | 47 | 12.1 (±1.6) | 92 | 12.5 (±1.5) | 63 | 13.3 (±1.2) | ns | **0.007** | **<0.0001** |
| Platelet Crit (%) | 267 | 0.201 (±0.07) | 271 | 0.215 (±0.07) | 47 | 0.2 (±0.1) | 92 | 0.2 (±0.08) | 63 | 0.2 (±0.08) | ns | ns | ns |
| Plate Large Cell Ratio (%) | 267 | 18.0 (±5.4) | 271 | 19.2 (±5.8) | 47 | 19.5 (±6.3) | 92 | 21.1 (±5.9) | 63 | 24.1 (±5.0) | ns | **0.008** | **<0.0001** |
| Plate Large Cell Count (unit/L) | 267 | 42.1 (±14.6) | 271 | 46.9 (±17.0) | 47 | 48.3 (±17.6) | 92 | 47.8 (±17.8) | 63 | 62.3 (±21.1) | ns | ns | **<0.0001** |

Data are presented as mean (± SD). Number of available results differs by each parameter due to limitations in blood sample volume for some participants. Two-sided paired t-test was conducted between V1 vs V2, V3 and V4 groups. *P* values in bold font are considered significant based on Bonferroni corrected *P* value = *P*<0.01667 (3 tests). Hepcidin (log), ferritin (log), sTfR (log), AGP (sqrt) and CRP (log) are all transformed to form a normal distribution before a two-sided paired t-test was completed.

**Supplemental Table 2: Comparison of iron status, inflammation and haematological parameters in cord venous and arterial blood.**

| **Sample Type** | **n** | **CDA** | **n** | **CDV** | ***CDA vs CDV***  ***(P value)*** |
| --- | --- | --- | --- | --- | --- |
| Serum Iron (µmol/L) | 255 | 22.7 (±6.9) | 255 | 22.7 (±7.0) | ns |
| TSAT (%) | 250 | 46.9 (±15.6) | 250 | 50.5 (±16.5) | **<0.0001** |
| Serum Hepcidin (ng/ml) | 248 | 19.7 (±14.4) | 248 | 19.6 (±14.6) | - |
| Log(Serum Hepcidin) | 248 | 2.7 (±0.7) | 248 | 2.7 (±0.8) | ns |
| UIBC (µmol/L) | 250 | 27.1 (±11.2) | 250 | 23.4 (±10.3) | **<0.0001** |
| TIBC (µmol/L) | 250 | 49.8 (±9.1) | 250 | 46.1 (±8.1) | **<0.0001** |
| Serum Ferritin (µg/L) | 254 | 277.0 (±235.0) | 254 | 215.7 (±161.1) | **-** |
| Log(Serum Ferritin) | 254 | 5.4 (±0.7) | 254 | 5.2 (±0.7) | **<0.0001** |
| Haemoglobin (g/dl) | 236 | 15.2 (±2.6) | 236 | 15.1 (±2.2) | ns |
| Haematocrit (%) | 237 | 43.0 (±7.5) | 237 | 42.4 (±6.7) | ns |
| Transferrin (g/L) | 257 | 2.0 (±0.3) | 257 | 2.0 (±0.3) | ns |
| Soluble Transferrin Receptor (mg/L) | 254 | 6.0 (±1.9) | 254 | 6.0 (±2.0) | - |
| Log(Soluble Transferrin Receptor) | 254 | 1.7 (±0.3) | 254 | 1.7 (±0.3) | ns |
| Haptoglobin (g/L) | 257 | 0.02 (±0.05) | 257 | 0.02 (±0.06) | ns |
| Serum AGP (g/L) | 257 | 0.2 (±0.1) | 257 | 0.2 (±0.1) | - |
| Sqrt(Serum AGP) | 257 | 0.4 (±0.2) | 257 | 0.4 (±0.2) | ns |
| Serum CRP (mg/L) | 257 | 0.2 (±0.5) | 257 | 0.2 (±0.5) | - |
| Log(Serum CRP) | 254 | -2.3 (±0.7) | 254 | -2.3 (±0.7) | ns |
| Mean Corpuscular Volume (fl) | 237 | 98.1 (±6.2) | 237 | 97.9 (±6.2) | ns |
| Mean Corpuscular Haemoglobin (pg) | 236 | 35.1 (±2.5) | 236 | 35.1 (±2.4) | ns |
| Mean Corpuscular Haemoglobin Concentration (g/dl) | 236 | 35.8 (±1.1) | 236 | 35.9 (±1.0) | ns |
| White Blood Cell Count (unit/L) | 235 | 14.0 (±5.8) | 235 | 13.4 (±5.8) | **0.004** |
| Lymphocyte Count (unit/L) | 235 | 5.4 (±3.4) | 235 | 4.8 (±3.6) | **<0.0001** |
| Lymphocyte Percentage (%) | 235 | 38.0 (±8.9) | 235 | 35.1 (±8.0) | **<0.0001** |
| MID Cell Count (unit/L) | 235 | 1.2 (±0.7) | 235 | 1.0 (±0.6) | **<0.0001** |
| MID Cell Percentage (%) | 235 | 8.8 (±3.2) | 235 | 8.1 (±2.4) | **0.0001** |
| Granulocyte Count (unit/L) | 235 | 7.5 (±2.9) | 235 | 7.5 (±2.7) | ns |
| Granulocyte Percentage (%) | 235 | 53.3 (±9.3) | 235 | 56.8 (±8.8) | **<0.0001** |
| Red Blood Cell Count (unit/L) | 237 | 4.4 (±0.8) | 237 | 4.3 (±0.7) | ns |
| Red Blood Cell Distribution Width (%) | 237 | 15.3 (±1.0) | 237 | 15.2 (±1.2) | ns |
| Red Blood Cell Distribution Width - Absolute (fl) | 237 | 81.4 (±8.9) | 237 | 80.9 (±9.0) | **0.008** |
| Platelet Count (unit/L) | 237 | 193.1 (±87.3) | 237 | 248.1 (±92.0) | **<0.0001** |
| Mean Platelet Volume (fl) | 231 | 8.7 (±0.8) | 231 | 8.3 (±0.8) | **<0.0001** |
| Platelet Distribution Width (%) | 231 | 44.6 (±3.5) | 231 | 43.4 (±3.0) | **<0.0001** |
| Platelet Distribution Width - Absolute (fl) | 231 | 12.4 (±1.5) | 231 | 11.8 (±1.2) | **<0.0001** |
| Platelet Crit (%) | 231 | 0.2 (±0.07) | 231 | 0.2 (±0.07) | **<0.0001** |
| Plate Large Cell Ratio (%) | 231 | 20.4 (±5.9) | 231 | 17.9 (±5.3) | **<0.0001** |
| Plate Large Cell Count (unit/L) | 231 | 37.9 (±15.1) | 231 | 42.5 (±14.7) | **<0.0001** |

Data are presented as mean (± SD) and analysed by two-sided paired t-test. p values in bold font are considered significant based on *P*<0.05. Hepcidin (log), ferritin (log), sTfR (log), AGP (sqrt) and CRP (log) are all transformed to form a normal distribution before a two-sided paired t-test was completed.

**Supplemental Figure 1: Weighted Pearson correlation network analysis of iron status and inflammation variables between cord and postnatal blood samples.**

**
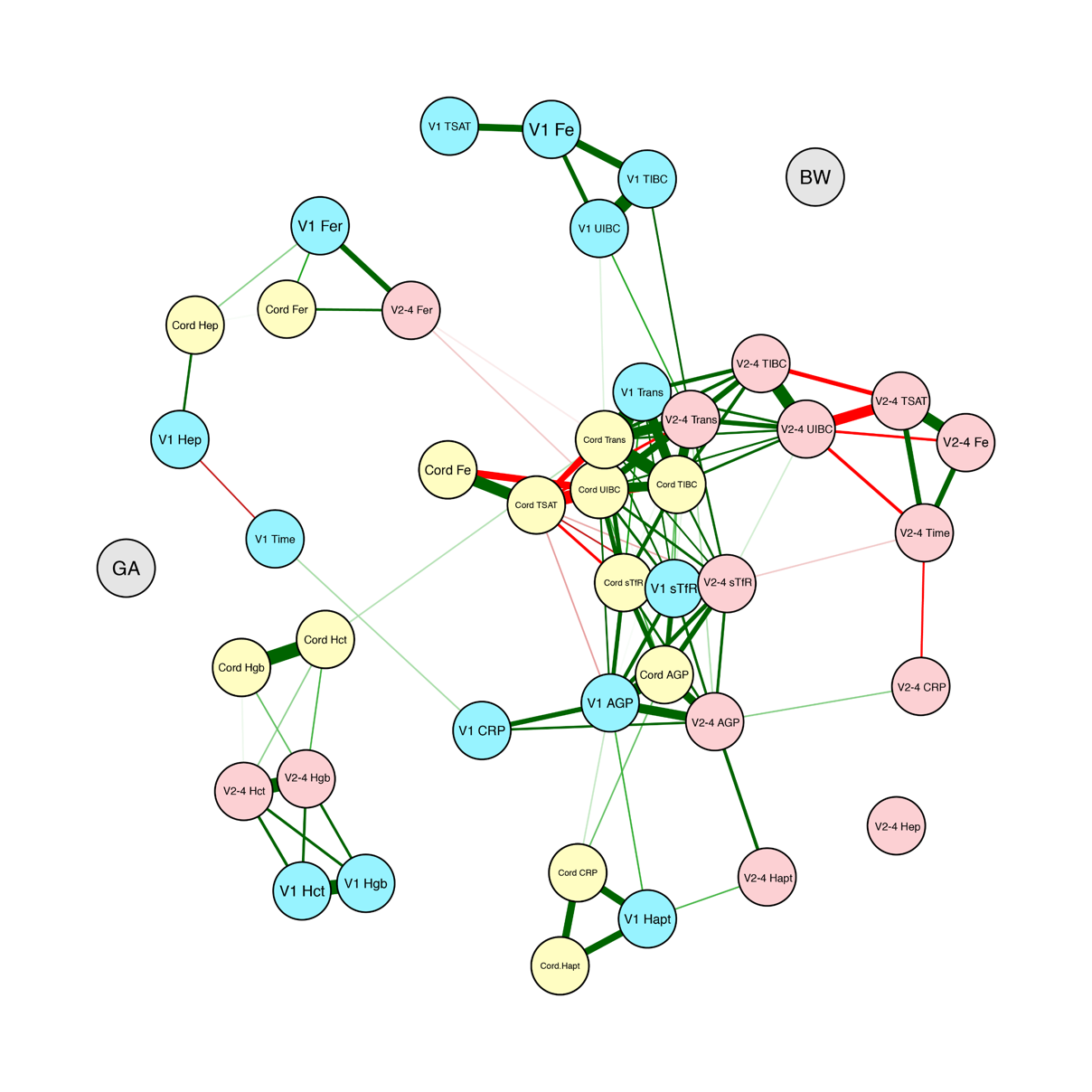
**

Produced using the Network App (see Methods). This analysis is formatted into the “spring” layout that uses the Fruchterman-Reingold Algorithm [26], placing the more strongly correlated nodes closer together. Node colours define sample type (YELLOW = CDV, BLUE = V1, PINK = V2-4).The direction and size of the Pearson correlation between two nodes is represented using the colour (RED = Negative, GREEN = Positive) and thickness of an edge. Cord = umbilical cord blood. V1 = venous blood from the dorsum of the hand at >6-≤24hrs. V2-4 = venous blood from the dorsum of the hand at >24-≤216hrs. Fer = ferritin, Hep = hepcidin, Hct = haematocrit, Hgb = haemoglobin, GA = gestational age, BW = birthweight, CRP = C-reactive protein, Hapt = haptoglobin, AGP = alpha 1-acid glycoprotein, sTfR = soluble transferrin receptor, UIBC = unbound iron-binding capacity, TIBC = total iron-binding capacity, Trans = transferrin, fe = serum iron, TSAT = transferrin saturation.

**Supplemental Figure 2: Comparison of iron status, inflammation and haematological parameters in cord arterial (CDA = BLUE) and venous (CDV = RED) blood.**

Box plots represent the arithmetic mean, with whiskers representing minimum and maximum values. **** = *P*<0.0001, *** = *P*<0.001, ** = *P*<0.01, * = *P*<0.05. No significance line = *P*>0·05.
